# Pro-inflammatory role of neutrophils populations in trauma patients: monitoring neutrophil populations

**DOI:** 10.3389/fimmu.2025.1565606

**Published:** 2025-07-08

**Authors:** Marcela Vlková, Julie Štíchová, Karolína Surá, Karolína Dvořáková, Vojtěch Kunčický, Ioanna Papatheodorou, Gabriela Blažková, Zuzana Tomášiková, Kamila Bendíčková, Ludmila Dohnálková, Alexandra Mýtniková, Jan Žák, Jan Kovařík, Milan Krtička, Tomáš Tomáš, Vladimír Šrámek, Martin Helán, Anna Kocurková, Jan Frič, Marcela Hortová-Kohoutková

**Affiliations:** ^1^ Institute of Clinical Immunology and Allergology, Faculty of Medicine, Masaryk University, Brno, Czechia; ^2^ Institute of Clinical Immunology and Allergology, St. Anne’s University Hospital, Brno, Czechia; ^3^ International Clinical Research Center, St. Anne’s University Hospital, Brno, Czechia; ^4^ Department of Biology, Faculty of Medicine, Masaryk University, Brno, Czechia; ^5^ Animal Physiology, Immunology and Developmental Biology, Faculty of Science, Masaryk University, Brno, Czechia; ^6^ International Clinical Research Center, Faculty of Medicine, Masaryk University, Brno, Czechia; ^7^ Department of Anesthesia and Intensive Care, St. Anne’s University Hospital, Brno, Czechia; ^8^ First Department of Chirurgic Surgery, St. Anne’s University Hospital and Faculty of Medicine, Masaryk University, Brno, Czechia; ^9^ Department of Trauma Surgery, University Hospital Brno, Brno, Czechia; ^10^ First Department of Orthopaedic Surgery, St. Anne’s University Hospital and Faculty of Medicine, Masaryk University, Brno, Czechia; ^11^ Department of Biophysics of Immune System, Institute of Biophysics of the Czech Academy of Sciences, Brno, Czechia; ^12^ Department of Modern Immunotherapy, Institute of Hematology and Blood Transfusion, Prague, Czechia

**Keywords:** trauma, neutrophils, SIRS, lactate, creatine kinase, TRISS, ISS

## Abstract

**Background:**

Trauma is a leading global cause of mortality, and systemic inflammatory response syndrome (SIRS) remains a significant complication, contributing to adverse outcomes. Neutrophils, as first responders to tissue injury, undergo substantial phenotypic and functional changes following trauma. This study investigates neutrophil subpopulations defined by CD16 and CD62L expression in trauma patients, focusing on their correlation with clinical biomarkers, trauma severity, and functional properties.

**Methods:**

We included 50 non-infectious trauma patients, categorized into SIRS and Non-SIRS groups, and 43 elective surgery patients as controls. Neutrophil subsets were analyzed at two time points (TP1 and TP2) using flow cytometry. Functional assays evaluated phagocytosis, oxidative burst, mitochondrial function, and degranulation. Correlations between neutrophil subpopulations and clinical markers, including lactate, creatine kinase, Injury Severity Score, and Trauma and Injury Severity Score, were examined.

**Results:**

Patients with SIRS exhibited higher proportions of banded neutrophils and CD16^low^CD62L^low^ neutrophils at TP1, alongside reduced levels of mature neutrophils. Elevated lactate and creatine kinase levels positively correlated with banded neutrophils and CD16^low^CD62L^low^ neutrophils, while negatively correlating with mature neutrophils CD16^high^CD62L^high^ and hypersegmented neutrophils CD16^high^CD62L^low^. Hypersegmented neutrophils were more prevalent in Non-SIRS patients at TP1 and in SIRS patients at TP2. Banded neutrophils showed a positive correlation with Injury Severity Score and an inverse correlation with Trauma and Injury Severity Score (TRISS), whereas hypersegmented neutrophils were negatively associated with ISS and positively correlated with TRISS. These correlations likely reflect the pro-inflammatory role of banded neutrophils and the inflammation-resolving function of hypersegmented neutrophils. CD16^low^CD62L^low^ neutrophils displayed impaired phagocytosis, oxidative burst, and degranulation capacity, indicating functional deficiencies.

**Conclusion:**

This study highlights the dynamic changes in neutrophil subpopulations in trauma and their association with systemic inflammation and clinical severity. Increased banded neutrophils correlate with SIRS and metabolic stress, whereas hypersegmented neutrophils may contribute to resolving inflammation. CD16^low^CD62L^low^ neutrophils exhibit functional impairments, warranting further investigation. Monitoring neutrophil subpopulations could aid in identifying trauma patients at risk for non-infectious SIRS and guide therapeutic interventions.

## Introduction

Trauma ranks among the ten leading causes of death worldwide and remains a significant public health challenge. While advancements in medical care have reduced trauma-related mortality (1, 2) caused by blood loss and coagulopathy, secondary complications such as systemic inflammatory response syndrome (SIRS) continue to pose considerable risks to patient outcomes (3). SIRS, characterized by a dysregulated cascade of pro-inflammatory cytokines and chemokines, is often accompanied by marked alterations in neutrophil phenotype and function (4–6).

Neutrophils, which constitute 50–70% of circulating white blood cells, are essential first responders to tissue injury. Their primary functions include the elimination of necrotic tissue and pathogens through mechanisms such as phagocytosis, degranulation, and the production of reactive oxygen species (ROS). Beyond their direct antimicrobial role, neutrophils actively participate in modulating immune responses by interacting with other immune cells and shaping the resolution or progression of inflammation (7).

Following trauma, emergency granulopoiesis results in the rapid release of heterogeneous neutrophils subsets from the bone marrow into circulation. These subsets include mature segmented neutrophils (CD16^high^CD62L^high^), immature banded neutrophils (CD16^low^CD62L^high^), and hypersegmented neutrophils (CD16^high^CD62L^low^) (8, 9). Additionally, a population of CD16^low^CD62L^low^ neutrophils has been identified as neutrophil progenitors in infants with bacterial and viral infections (10). While these cells are believed to represent immature neutrophil forms, their functional roles in trauma-related immune responses remain to be elucidated.

Neutrophil dysfunction in trauma has significant clinical implications. Dysregulated neutrophil activation and recruitment can exacerbate tissue damage, prolong inflammation, and contribute to the development of severe complications such as sepsis and multiorgan failure (11–13). Specific subpopulations, such as hypersegmented neutrophils have been associated with immunoregulatory roles, including the ability to regulate pro-inflammatory T-lymphocyte activity through Mac-1 receptors (3). Furthermore, the early mobilization of banded neutrophils has been proposed as a potential biomarker for trauma severity and immune response dynamics (9).

Understanding the heterogeneity of neutrophil subsets and their functional properties is crucial for advancing our knowledge of trauma-related immune dysregulation. In trauma patients, the expression of neutrophil activation markers such as CD10, CD11b, the degranulation marker CD66b, and chemotaxis markers CD181 and CD182 is frequently discussed (3, 7). While recent years have seen the classification of neutrophils into subsets based on CD16 and CD62L expression, the baseline expression of these markers in these subsets, as well as how their levels change upon activation, has not yet been clearly defined. In this study, we present for the first time the correlations between neutrophil subsets, clinical biomarkers such as lactate and creatine kinase, and trauma severity scores, including the Trauma and Injury Severity Score (TRISS) and Injury Severity Score (ISS). Subsequently, we focused on the functional properties of CD16^low^CD62L^low^ neutrophils in trauma patients, analyzing their phagocytic capacity, oxidative burst activity, changes in activation markers (CD10, CD11b), the degranulation marker CD66b, and chemotaxis markers CD181 and CD182. Additionally, we examined the mitochondrial content, membrane potential, and mitochondrial ROS production on defined neutrophil populations. Through this integrative approach, we aim to elucidate the role of neutrophils in trauma-related inflammation and their potential impact on clinical outcomes.

## Methods

### Study participants

All participants in this study were patients suffering from combined injury or blunt polytrauma admitted to either high threshold Emergency of the Department of Anesthesiology and Intensive Care of St. Anne’s University Hospital in Brno (n=38) or the Clinic of Accident Surgery of the University Hospital Brno (n=12), Czech Republic. Fifty trauma patients (22 females, 28 males; median age 43, range 19–78 years) were enrolled. At the time of admission to the Intensive Care Unit (ICU), none of the patients showed signs of infection. Based on the clinical and laboratory evaluation during the first 24 hours of hospitalization, patients were categorized into two groups: the SIRS group (Systemic Inflammatory Response Syndrome; n=16) and the Non-SIRS group (n=34). Patients in the SIRS group exhibited at least two of the following circumstances: tachycardia, hypotension, need for oxygen therapy, lactate elevation, or oliguria. Detailed clinical characteristics of the study population are presented in **Table 1**.

**Table 1:** Clinical and laboratory characteristics of patients.

Demographics and Nature of trauma (N)		Non-SIRS	Standard deviation	SIRS	Standard deviation	p (Non-SIRS/SIRS)
Number of patients		**38**		**12**		
Male/Female (N)		**22/12**		**12/2**		
Age (years)		**52,3**	*17,8*	**49,6**	*15,7*	ns
BMI		**26**	*4,1*	**27,2**	*4,5*	ns
Motor-vehicle accident		**32**		**6**		
Fall from a height		**6**		**5**		
Other blunt injury		**0**		**1**		
Mortality due to MODS		**1**		**1**		

Laboratory parameters	Normal range	Median	Min - Max	Median	Min - Max	p (Non-SIRS/SIRS)
Leukocytes (*10*9/l)	*4,0 - 10,0*	**11,05**	*7,02 - 26,6*	**15,1**	*11,21 - 31,14*	**0.0336**
Immature granulocytes (%)	*0,0 - 0,6*	**0,3**	*0,2 - 1,5*	**0,5**	*0,4 - 0,8*	ns
C-reactive protein CRP (mg/l)	*0,0 - 5,0*	**1,15**	*0,6 - 39,0*	**1,5**	*0,6 - 13,7*	ns
Lactate (mmol/l)	*0,5 - 2,2*	**1,4**	*0,6 - 4,2*	**2,1**	*1,4 - 5,9*	**0.0162**
Thrombocytes (*10*9/l)	*150 - 400*	**234**	*90 - 674*	**256,5**	*150 - 355*	ns
Hemoglobin (g/l)	*120 - 175*	**128,0**	*84 - 164*	**134,5**	*98 - 169*	ns
Creatine kinase (ukat/l)	*0,0 - 3,2*	**4,04**	*1,6 - 21,4*	**7,1**	*3,9 - 43,3*	**0.0008**
Myoglobin (ug/l)	*25,0 - 72,0*	**231,1**	*31,6 - 2864*	**713,0**	*279,5 - 1466*	**0.0491**
Alcohol (‰)	*0*	**0**	*0 - 2,5*	**0,0**	*0 - 1,8*	ns
Injury Severity Score (ISS 0-75)		**19,5**	*4 - 57*	**36,0**	*17 - 66*	**0.0037**
Glasgow Coma Scale (GCS 3-15)		**15**	*14 - 15*	**15,0**	*3 - 15*	**0.0443**
TRISS Score - % of survival		**96,75**	*53,45 - 99,61*	**86,7**	*2,11 - 98,43*	**0.0003**

Demographic overview of the group of 50 trauma patients, including the cause of trauma and the evaluation of measured laboratory parameters upon hospital admission. The percentage of immature neutrophils represents the percentage of immature neutrophils measured using a hematology analyzer. The table also includes an assessment of scoring systems for both the SIRS and Non-SIRS patient groups. Statistically significant results are highlighted in bold, with a significance level of p < 0.05. Data were tested for normal distribution; for normally distributed data, a Student's t-test was applied, while non-normally distributed data were analyzed using the Mann-Whitney U test.

Blood samples were collected from all patients at two specific time points. The first sample (TP1) was obtained shortly after ICU admission, with a mean interval of 20.8 hours (SD ± 9.6 hours). The second sample (TP2) was collected on average 4 days later (SD ± 1 day). All blood samples were processed within 1 hour of collection. The control group consisted of 43 patients scheduled for hip or knee joint replacement surgery (27 females, 16 males; median age 71, range 43–91 years).

### Flow cytometry characterization of neutrophils

Neutrophils were characterized using a combination of fluorescently labeled human monoclonal antibodies: CD15 FITC (clone MEM-158), CD193 PE (clone 5E8), CD45 PE-DY647 (clone MEM-28), CD14 PerCP Cy5.5 (clone MEM-15), CD62L PC7 (clone LT-TD180), CD10 APC (clone MEM-78), CD11b PB (clone ICRF44), and CD16 PO (clone 3G8) (all antibodies from Exbio Praha, Vestec, Czech Republic). Additional antibodies included CD181 APC (clone 8F1/CXCR1), CD182 APC (clone 5E8/CXCR2), and CD66b Alexa Fluor 700 (clone 10F5; Sony Biotechnology, San Jose, CA, USA). Fresh whole blood samples were incubated with monoclonal antibodies for 30 minutes at 4°C in the dark. Erythrocytes were lysed using formic acid, followed by the addition of a stop solution. The samples were washed with phosphate-buffered saline (PBS) before analysis. Data acquisition was performed on a Navios flow cytometer (10 colors, 3 lasers; Beckman Coulter, Miami, FL, USA), and data were analyzed using the Kaluza software (Beckman Coulter, Brea, CA, USA).

### Oxidative burst of neutrophils

To assess the oxidative burst of neutrophils, mean fluorescence intensity (MFI) of rhodamine 123 was measured using flow cytometry. Two tubes of whole blood were prepared per patient: one unstimulated and one stimulated with opsonized 1 × 10^7^ *Staphylococcus aureus* (Wood strain without protein A) BioParticles™ (Invitrogen™, Life Technologies Corporation, Oregon, USA). Each tube contained 100 µl of blood and 10 µl of diluted Dihydrorhodamine 123 (final concentration 375 ng/ml; Sigma-Aldrich, St. Louis, MO, USA). Samples were incubated for 30 minutes at 37°C, washed with PBS, and incubated for another 30 minutes at 4°C with monoclonal antibodies: CD14 PerCP Cy5.5, CD66b Alexa Fluor 700, CD11b PB, CD10 PC7 (clone MEM-78), CD62L APC (clone LT-TF180), CD16 APC-Cy7 (clone 3G8) and CD45 PO (clone HI30),(all antibodies from Exbio Praha). Erythrocytes were lysed by formic acid as mentioned above and samples were measured using a Navios flow cytometer.

### Phagocytosis assay

Phagocytic activity of neutrophils was assessed using FITC-labeled *Escherichia coli* strain K12. (IngoFlowEx kit; Exbio Praha). Whole blood samples were divided into two tubes: one unstimulated and one stimulated with 10 µl of FITC-labeled *E. coli*. Following a 30 minute incubation at 37°C, samples were washed with PBS, labeled with monoclonal antibodies, (CD14 PerCP Cy5.5, CD66b Alexa Fluor 700, CD11b PB, CD10 PC7, CD62L APC, CD16 APC-Cy7 and CD45 PO) lysed, and analyzed using a Navios flow cytometer.

### Evaluation of mitochondrial functionality

Mitochondrial functionality was assessed using specific fluorescent probes: MitoTracker™ Green FM (MTG) for mitochondrial content and MitoTracker™ Red CMXRos (MTR) for mitochondrial membrane potential (all probe, Invitrogen™) (14). Blood samples (100 µl) were incubated with these probes along with monoclonal antibodies (CD193 PE, CD62L PC7, CD64 APC, CD11b PB, CD16 PO, CD66b Alexa Fluor 700). Mitochondrial ROS production was evaluated using MitoSOX Red (MTS) in pre-lysed blood samples incubated at either 4°C (unstimulated) or 37°C (stimulated with *Staphylococcus aureus*). Following an incubation, samples were washed with PBS, labeled with monoclonal antibodies and lysed. Data were acquired on a Navios flow cytometer and analyzed with the Kaluza software.

### Statistical analysis

All statistical analyses were performed using GraphPad Prism 5 (GraphPad Software Inc., California, USA). Data normality was assessed using the Shapiro-Wilk test. Non-parametric tests, including the Mann-Whitney U test, Wilcoxon signed-rank test, and Kruskal-Wallis test with Dunn’s correction for multiple comparisons, were employed as appropriate. Correlations were analyzed using Spearman’s rank correlation test. Results were considered statistically significant at p < 0.05.

## Results

A total of 50 non-infectious trauma patients were included in the study. Based on their clinical and laboratory presentation, 16 patients were categorized as trauma wit SIRS (TRA with SIRS), while the remaining 34 were designated as TRA without SIRS (Non-SIRS). None of the patients displayed signs of infection upon admission. The clinical characteristics of the study population are summarized in **Table 1**. Initial laboratory assessments revealed that SIRS patients exhibited significantly higher leukocyte counts, as well as elevated serum lactate and creatine kinase (CK) levels, compared to Non-SIRS patients. Interestingly, despite the lower ISS observed in the SIRS group, these patients demonstrated lower Glasgow Coma Scale (GCS) scores and Trauma and Injury Severity Scores (TRISS) compared to Non-SIRS patients (**Table 1**).

Blood samples were collected from trauma patients at two distinct time points (TP1 and TP2) to assess dynamic changes in neutrophil subpopulations and other parameters, allowing for the monitoring of immediate and evolving immune response following trauma. The control group consisted of 43 individuals undergoing elective hip or knee joint replacement surgery, who were used as a reference for baseline neutrophil characteristics.

### Neutrophil subpopulations in trauma patients

**Figure 1:**
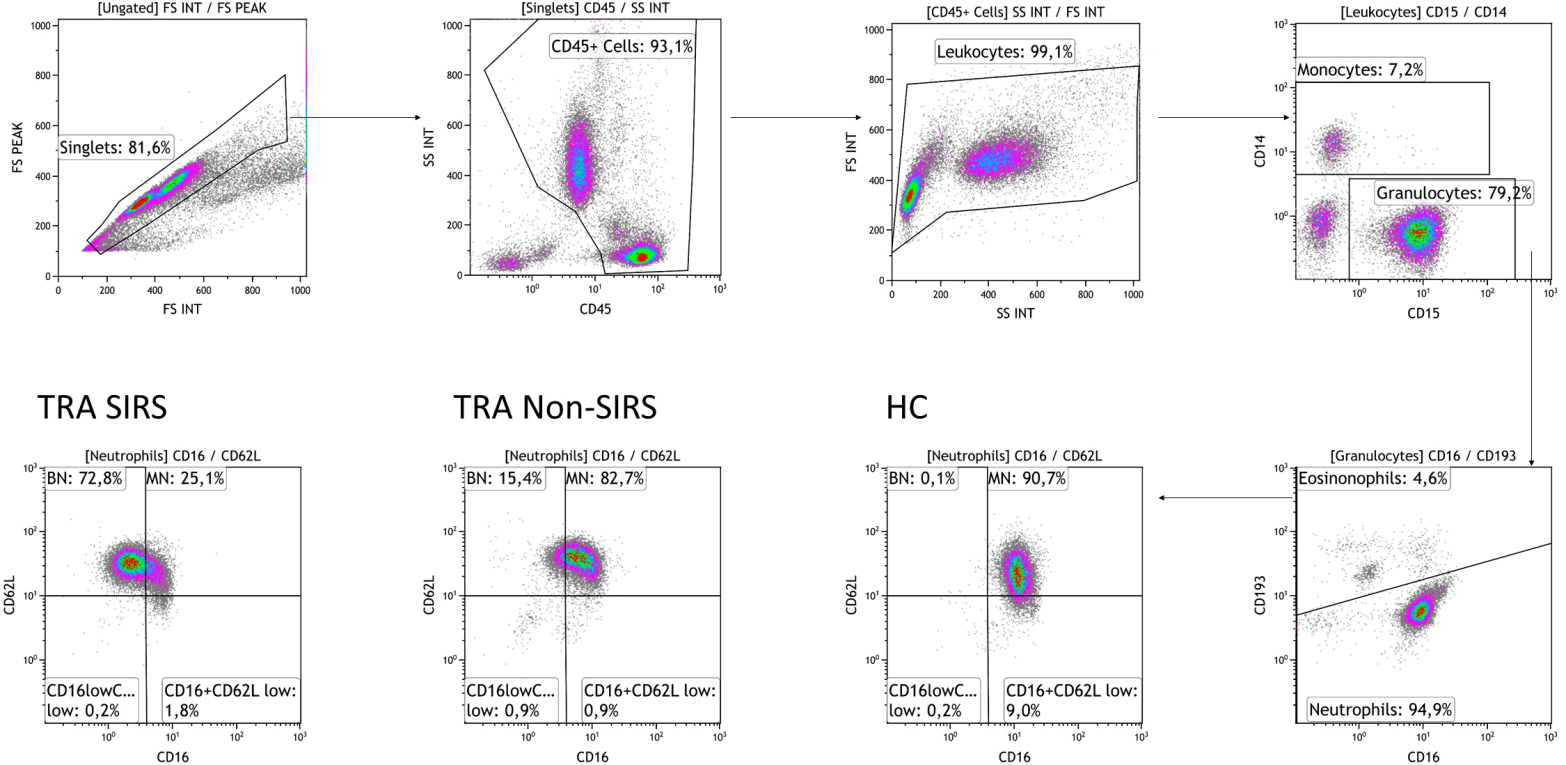
Plots illustrating the gating strategy for neutrophil subpopulations. The initial gate was set on FSC-A and FSC-H to exclude doublets, followed by gating on CD45^high^ to eliminate debris, and FSC-H and SSC to exclude degranulated cells. Neutrophils were identified as CD45^high^CD14^low^CD15^high^CD193^low^ cells and further classified into subpopulations based on surface expression of CD62L and CD16: CD16^high^CD62L^high^ mature neutrophils (MN), CD16^low^CD62L^high^ banded neutrophils (BN), CD16^high^CD62L^low^ hypersegmented neutrophils, and CD16^low^CD62L^low^ neutrophils. Representative data from one healthy donor (HD), one Non-SIRS patient, and one SIRS patient were analyzed using the Kaluza software.

**Figure 2:**
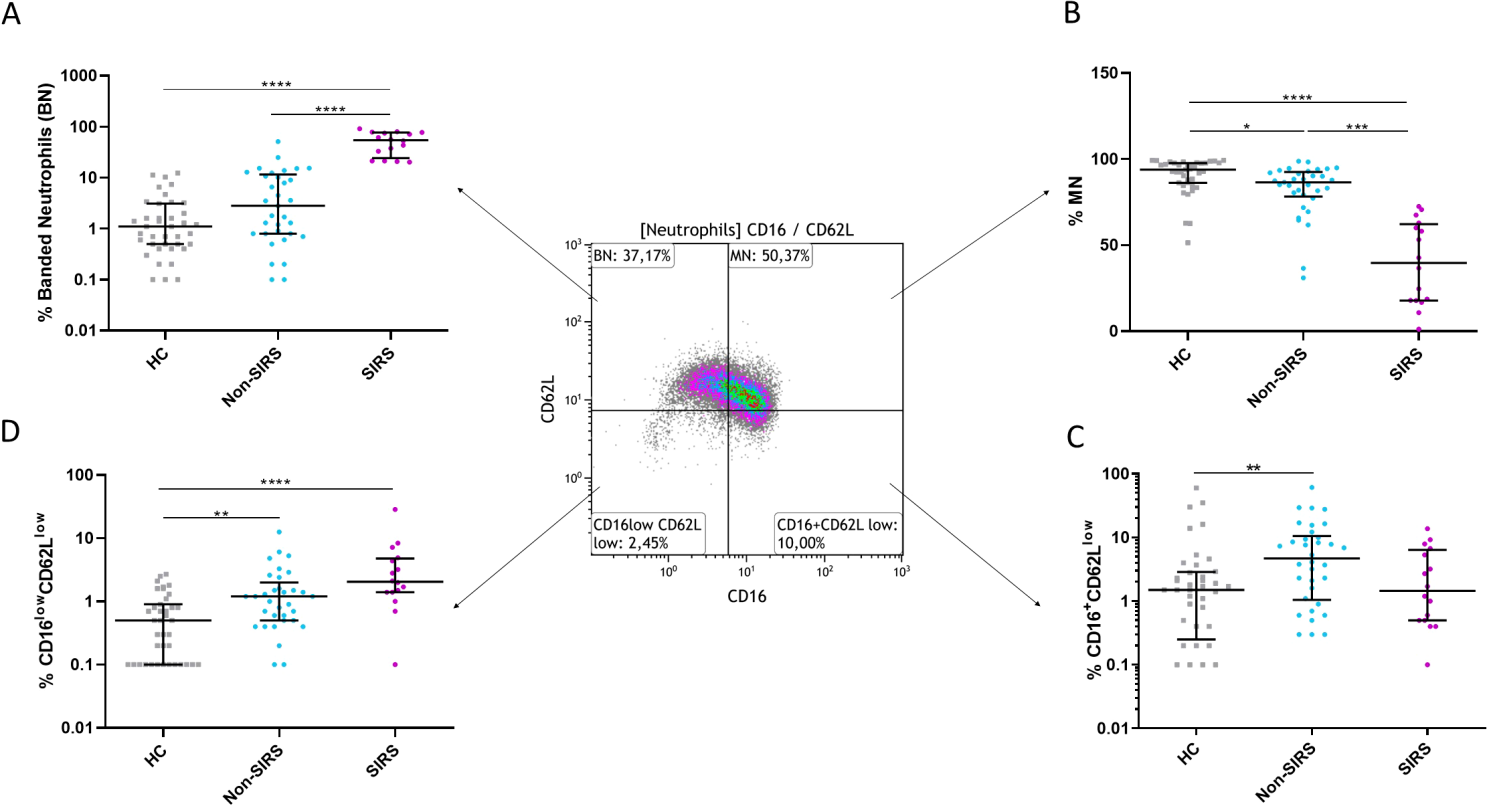
The percentages of neutrophil subpopulations for Non-SIRS patients, SIRS patients, and healthy controls. The percentage of **(A)** banded neutrophils (BN), **(B)** mature neutrophils (MN), **(C)** CD16^high^CD62L^low^ hypersegmented neutrophils, and **(D)** CD16^low^CD62L^low^ neutrophils. Data were non-normally distributed (Shapiro-Wilk test), and statistical analysis was conducted using the Kruskal-Wallis test with Dunn’s correction for multiple comparisons (all vs. all); *p ≤ 0.05, **p ≤ 0.01, ***p ≤ 0.001, ****p ≤ 0.0001.Sample sizes: N = 39 healthy controls, 33 Non-SIRS patients, and 16 SIRS patients.

To identify and characterize neutrophil subpopulations, we utilized the markers CD16 and CD62L. Neutrophils were gated based on their granularity while excluding eosinophils (gating strategy shown in **Figure 1**). Subpopulations included CD16^low^CD62L^high^ (banded neutrophils, BN), CD16^high^CD62L^high^ (mature neutrophils, MN), CD16^high^CD62L^low^ (hypersegmented neutrophils HSN) and CD16^low^CD62L^low^ neutrophils (Pillay J et al., 2012). Analysis of data collected at TP1 revealed a significant reduction in mature neutrophils (MN) in both trauma groups, with a more pronounced decrease in SIRS patients. Conversely, an significantly increase in BN was observed, particularly in SIRS patients (**Figures 2A, B**). The decline in MN among SIRS patients was primarily attributed to a rise in CD16^low^CD62L^low^ neutrophils, while in Non-SIRS patients, the reduction also involved HSN neutrophils (**Figures 2C, D**).

**Figure 3:**
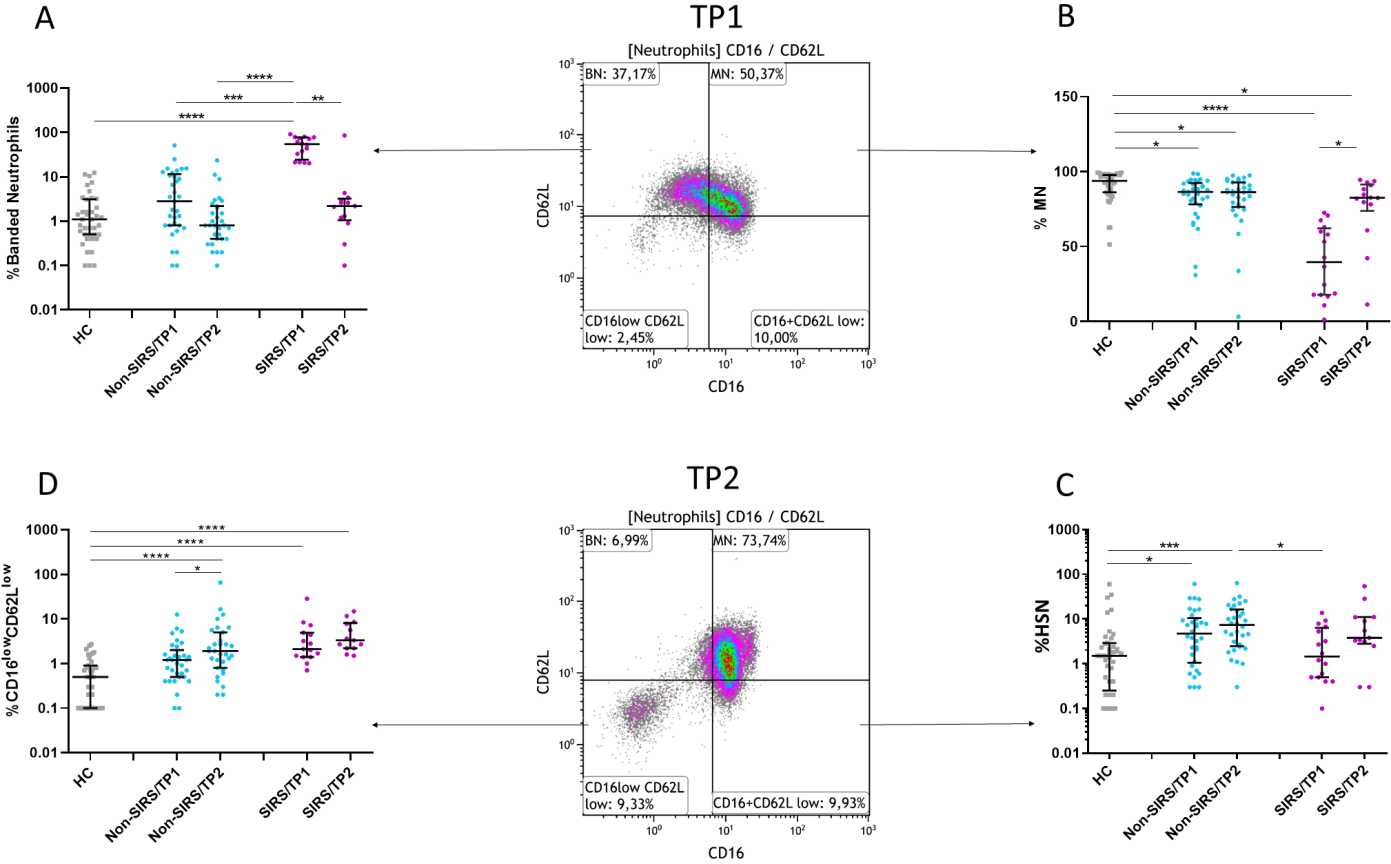
Frequency of neutrophil subpopulations at two time points (TP1 and TP2). **(A, B)** The frequency of mature neutrophils (MN) increases, and the frequency of banded neutrophils (BN) decreases in SIRS patients at TP2. **(C)** The frequency of hypersegmented neutrophils (HSN) increases in Non-SIRS patients at TP1 and TP2. **(D)** The frequency of CD16^low^CD62L^low^ neutrophils increases in Non-SIRS patients at TP2 and remains elevated in SIRS patients. Data were non-normally distributed (Shapiro-Wilk test), and statistical analysis was conducted using the Kruskal-Wallis test with Dunn’s correction for multiple comparisons (all vs. all); *p ≤ 0.05, **p ≤ 0.01, ***p ≤ 0.001, ****p ≤ 0.0001. Sample sizes: TP1: N = 39 healthy controls, 33 Non-SIRS patients, 16 SIRS patients; TP2: N = 31 Non-SIRS patients, 13 SIRS patients.

Although trauma patients exhibited elevated absolute neutrophil counts compared to controls, no significant differences were observed between SIRS and Non-SIRS patients in total leukocyte or neutrophil counts (**Supplementary Figures 1A–C**). The analysis of neutrophil subpopulations at TP2 revealed a normalization of BN and an increase in mature neutrophils MN in SIRS patients, while Non-SIRS patients showed an increase in both HSN and CD16^low^CD62L^low^ neutrophils (**Figures 3A–D**). In the overall study cohort, a significant correlation was found between patient age and the percentage of mature neutrophils, (R = –0.2858, p = 0.0443). This association was also confirmed in non-SIRS patients, (R = –0.3614, p = 0.0358), but did not reach statistical significance in the SIRS subgroup (R = –0.496, p = 0.0526, Spearman test). No significant correlations with age or associations with sex were identified for the remaining neutrophil populations.

### Correlation of neutrophil subpopulations with creatine kinase and lactate levels

In SIRS patients, elevated levels of CK and lactate were observed at the time of hospital admission (**Table 1**). Therefore, we investigated whether these markers were associated with the proportional representation of specific neutrophil subpopulations. Serum CK levels, a marker of muscle injury, were negatively correlated with the percentages of MN and HSN at TP1. In contrast, a positive correlation was observed between CK levels and the percentage of BN at TP1, as well as CD16^low^CD62L^low^ neutrophils at TP2 (**Table 2**).

**Table 2:** Correlation of neutrophil subpopulations with creatine kinase and lactate levels.

Correlation coefficient and probability	Creatine kinase (ukat/l)		Lactate (mmol/l)		Lactate (mmol/l)	
	ICU admission		ICU admission		TP1	
	r	p	r	p	r	p
% MN/TP1	**-0,332**	**0.0416**	*-0,269*	*ns*	**-0,792**	**0.0019**
% BN/TP1	**0,473**	**0.0027**	**0,427**	**0.0028**	**0,823**	**0.0009**
%CD16+CD62Llow /TP1	**-0,378**	**0.0195**	*-0,265*	*ns*	**-0,638**	**0.0215**
CD16lowCD62Llow/TP1	*0,066*	*ns*	**0,377**	**0.009**	0,131	*ns*
% MN/TP2	*-0,122*	*ns*	*-0,177*	*ns*	*-0,360*	*ns*
% BN/TP2	*0,146*	*ns*	*0,005*	*ns*	**0,657**	**0.0318**
%CD16+CD62Llow /TP2	*-0,276*	*ns*	*0,010*	*ns*	*-0,242*	*ns*
CD16lowCD62Llow/TP2	**0,359**	**0.0401**	**0,337**	**0.0292**	*0,055*	*ns*

The table presents Spearman correlation coefficients for non-parametric correlations between creatine kinase concentrations and specific neutrophil populations at TP1 and TP2, as well as between lactate concentrations and neutrophil populations at TP1 and TP2. Statistically significant results are highlighted in bold. Sample sizes: Creatine kinase: N = 37 TRA patients; Lactate: N=47 TRA patients. r (Spearman correlation coefficients); ns (not significant).

Next, we analyzed the association between serum lactate concentrations, indicative of metabolic stress, anaerobic metabolism and tissue hypoxia and the distribution of neutrophil subpopulations. Higher lactate concentrations correlated positively with the percentages of immature neutrophils and CD16^low^CD62L^low^ subsets at both TP1 and TP2. In contrast, MN and CD16^high^CD62L^low^ subsets were negatively correlated with lactate levels at TP1 (**Table 2**).

### Neutrophil subpopulations and clinical severity scores

Patients with SIRS exhibited a higher ISS and lower TRISS scores compared to Non-SIRS patients, reflecting increased trauma severity and reduced survival probability (**Table 1**). To further address the potential role of neutrophil subpopulations in SIRS, we analyzed their associations between Injury Severity Score and Trauma and Injury Severity Score. The percentage of BN at TP1 was inversely correlated with the TRISS score and positively associated with ISS (**Table 3**). CD16^low^CD62L^low^ neutrophils at TP1 showed a weaker inverse correlation with the TRISS score and no significant association with ISS. At TP2, BN maintained a weaker inverse correlation with TRISS and a positive association with ISS (**Table 3**).

**Table 3:** Neutrophil subpopulations and clinical severity scorest.

Correlation coefficient and probability	TRISS score		Injury severity score	
	r	p	r	p
c lactate (mmol/l)	*-0,252*	*ns*	*0,279*	*ns*
c kreatin kinase (µkat/l)	*0,1975*	*ns*	** 0,401**	**0.0126**
% MN/TP1	**0,455**	**0.0009**	**-0,284**	**0.0457**
% BN/TP1	**-0,541**	**<0.0001**	**0,541**	**<0.0001**
% CD16+CD62L low/TP1	*0,092*	*ns*	**-0,459**	**0.0008**
% CD16low CD62L low/TP1	**-0,357**	**0.0109**	*0,262*	*ns*
% MN/TP2	*0,005*	ns	*0,044*	*ns*
% BN/TP2	**-0,323**	**0.0306**	*0,005*	*ns*
% CD16+CD62L low/TP2	*0,183*	*ns*	*0,114*	*ns*
% CD16low CD62L low/TP2	*-0,124*	*ns*	*0,221*	*ns*

The table presents Spearman correlation coefficients for non-parametric correlations between Trauma and injury severity score (TRISS) and specific neutrophil populations at TP1 and TP2, as well as between Injury severity score (ISS) and specific neutrophil populations at TP1 and TP2. Statistically significant results are highlighted in bold. Sample sizes: Both TRISS and ISS: N = 50 TRA patients. r (Spearman correlation coefficients); ns (not significant).

### Functional analysis of neutrophil subsets

**Figure 4:**
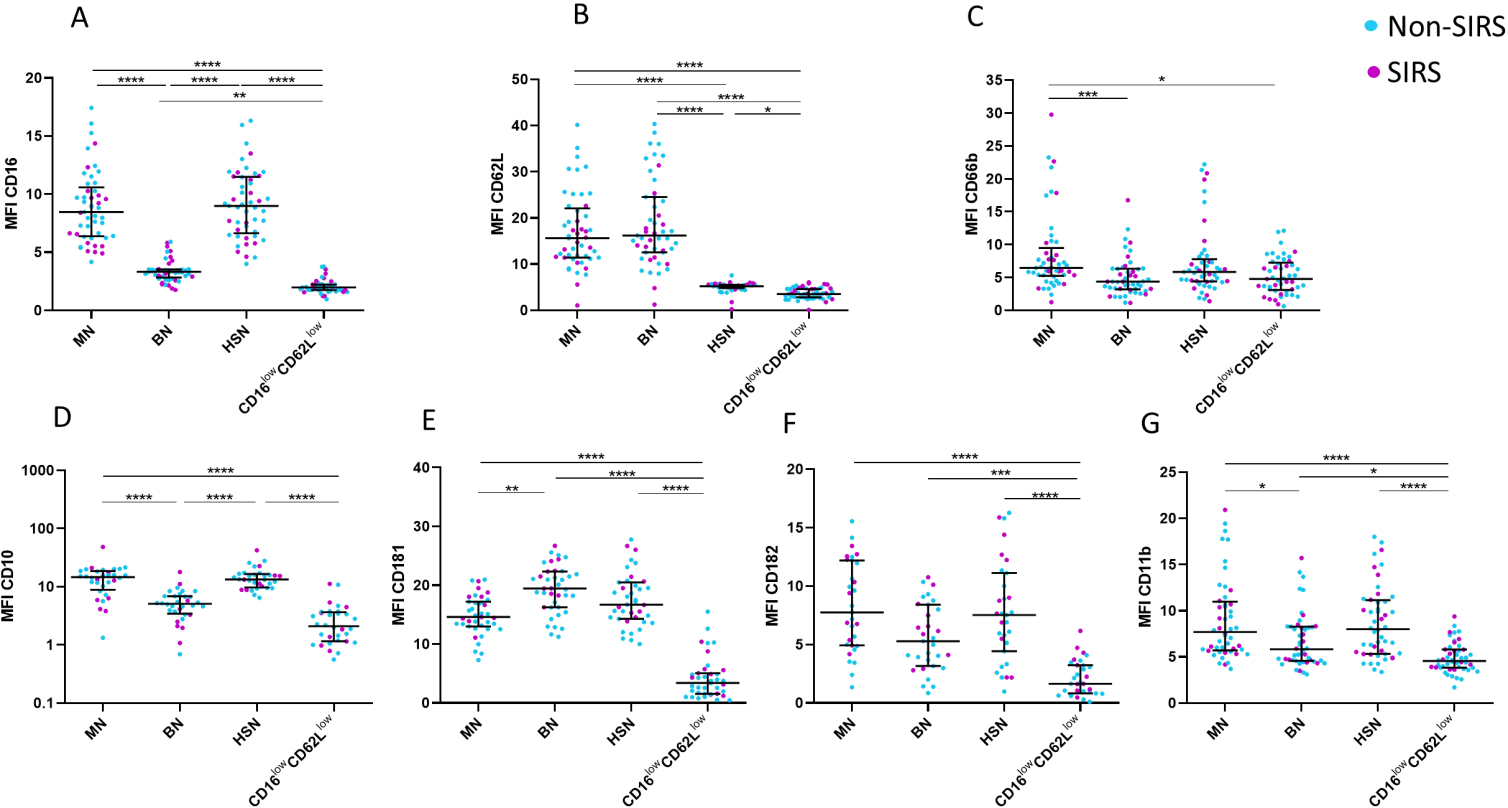
CD marker expression for individual neutrophil subpopulations. Cell surface expression of neutrophil markers on different neutrophil subpopulations at TP1, including **(A)** CD16, **(B)** CD62L, **(C)** CD66b, **(D)** CD11b, **(E)** CD10, **(F)** CD181, and **(G)** CD182. CD16^low^CD62L^low^ neutrophils exhibit low expression of all analyzed markers. Decreased expression of CD10 and CD11b on banded neutrophils (BN). Data were non-normally distributed (Shapiro-Wilk test), and statistical analysis was conducted using the Kruskal-Wallis test with Dunn’s correction for multiple comparisons (all vs. all); *p ≤ 0.05, **p ≤ 0.01, ***p ≤ 0.001, ****p ≤ 0.0001. Sample size CD16, CD62L CD66b and CD11b: TP1: N = 33 Non-SIRS patients, 16 SIRS patients; CD10: TP1: N = 32 Non-SIRS patients, 9 SIRS patients; CD181: TP1: N = 39 Non-SIRS patients, 11 SIRS patients; CD182: TP1: N = 31 Non-SIRS patients, 12 SIRS patients.

**Figure 5:**
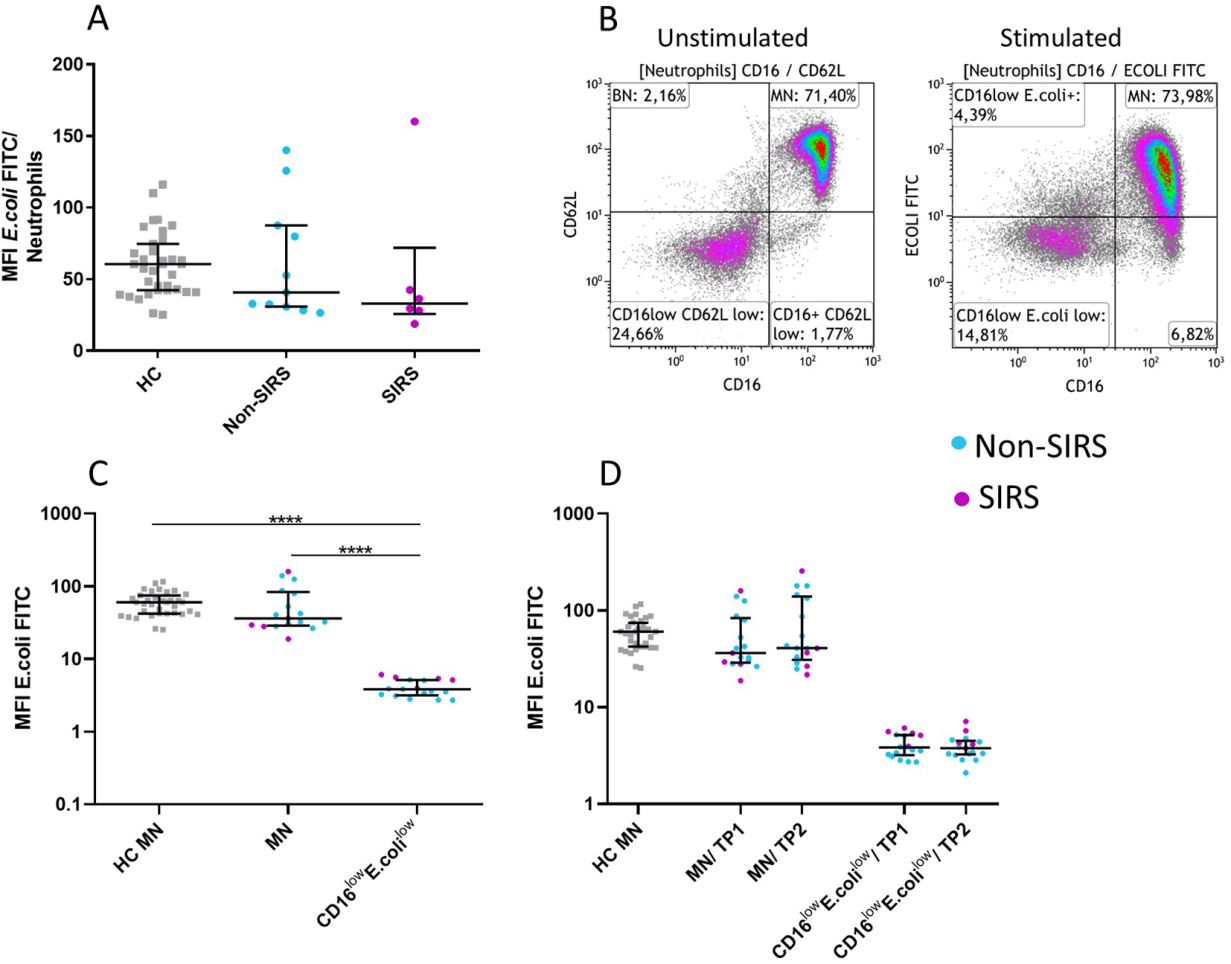
Neutrophil Functional Test: Ingestion capacity in neutrophil subpopulations of FITC-labeled *E. coli*. Normal ingestion capacity of FITC-labeled *E. coli* in Non-SIRS and SIRS patients at TP1. **(A)** CD16^low^CD62L^low^ neutrophils exhibit reduced ingestion capacity of *E. coli* **(B, C)** compared to mature neutrophils (MN). The functional properties of both populations remained unchanged over time (TP1 and TP2) **(D)**. The data are presented as the mean fluorescence intensity of FITC-labeled *E. coli*. Data were non-normally distributed (Shapiro-Wilk test), and statistical analysis was conducted using the Kruskal-Wallis test with Dunn’s correction for multiple comparisons (all vs. all); ****p ≤ 0.0001. Sample sizes: Ingestion of *E. coli* (MFI *E. coli* FITC): TP1, TP2: N = 35 healthy controls, 12 Non-SIRS patients, 6 SIRS patients.

**Figure 6:**
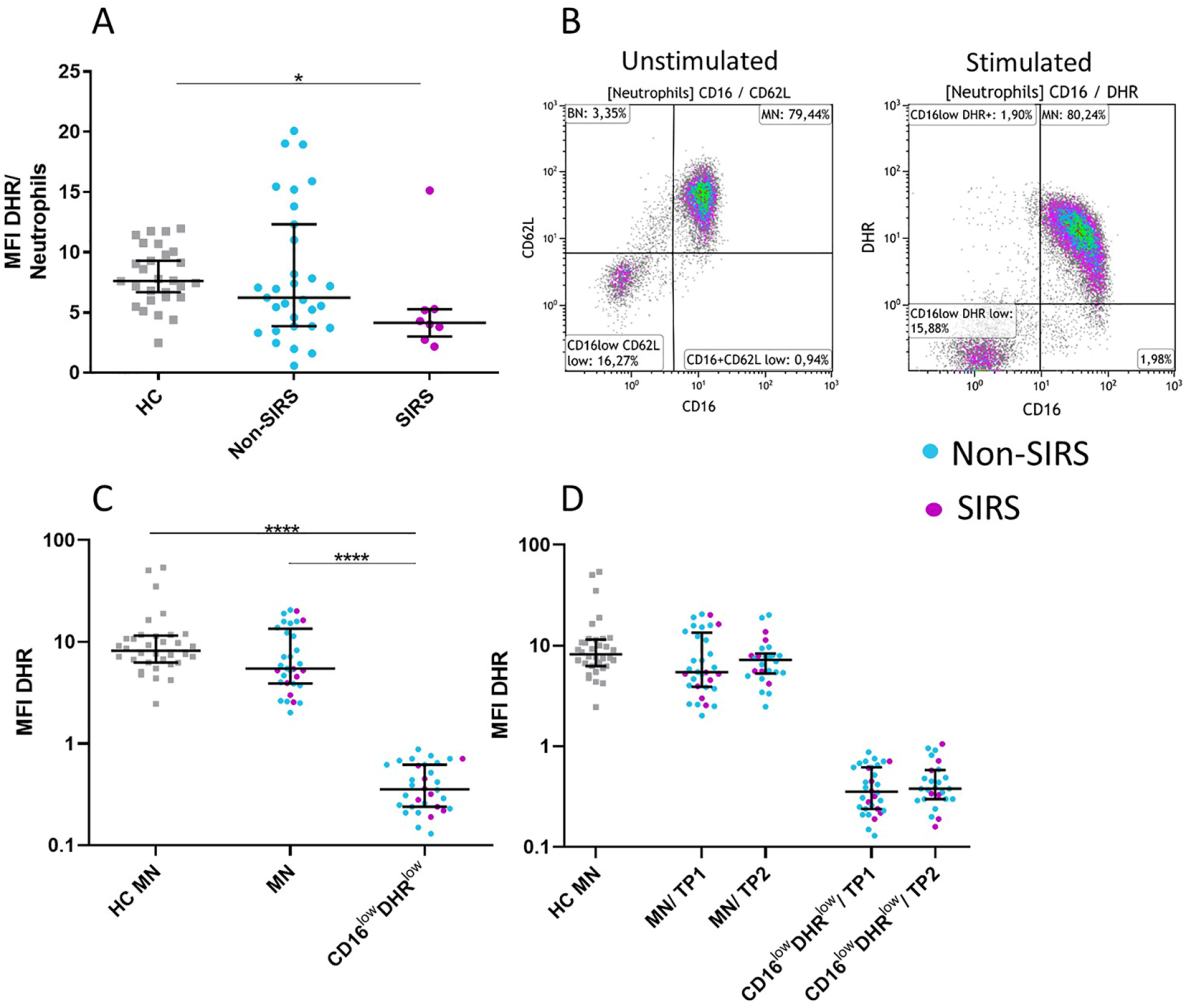
Neutrophil functional test: measurement of oxidative burst in neutrophil subpopulations following *Staphylococcus aureus* stimulation. Reduced oxidative burst capacity in neutrophils from SIRS patients was observed. **(A)** The analysis compared the mean fluorescence intensity of rhodamine (MFI DHR) in total neutrophils following *Staphylococcus aureus* (SA) stimulation. CD16^low^CD62L^low^ neutrophils demonstrate a reduced oxidative burst response to SA stimulation **(B–D)** compared to mature neutrophils (MN). The data are presented as the mean fluorescence intensity of rhodamine (MFI DHR) following *Staphylococcus aureus* stimulation. The functional properties of both populations remained unchanged over time (TP1 and TP2) **(B, C)**. Data were non-normally distributed (Shapiro-Wilk test), and statistical analysis was conducted using the Kruskal-Wallis test with Dunn’s correction for multiple comparisons (all vs. all); *p ≤ 0.05, ****p ≤ 0.0001. oxidative burst (MFI DHR): TP1: N = 29 healthy controls, 24 Non-SIRS patients, 8 SIRS patients; TP2: N = 29 healthy controls, 20 Non-SIRS patients, 6 SIRS patients.

Phenotypic analysis of neutrophil subpopulations, defined based on CD16 and CD62L expression, demonstrated that BN exhibit reduced expression of CD10, CD11b, and CD16, along with increased expression of CD181. CD16^low^CD62L^low^ neutrophils exhibited low expression of neutrophil activation markers CD10, CD11b, CD181, CD182 suggesting potential functional impairments (**Figure 4**; **Supplementary Figure 2**). Therefore, we assessed their phagocytic capacity using FITC-labeled *E. coli* particles and oxidative burst activity. The total phagocytic capacity of neutrophils revealed no significant differences between trauma patients and controls. (**Figure 5A**). However, CD16^low^CD62L^low^ neutrophils displayed reduced phagocytic activity in comparison to MN (**Figures 5B, C**). ROS production by neutrophils upon stimulation with *Staphylococcus aureus* (SA) was diminished in SIRS patients compared to Non-SIRS patients and controls (**Figure 6A**). Similarly, CD16^low^CD62L^low^ neutrophils exhibited a significantly lower capacity for ROS production compared to MN a pattern observed consistently at both TP1 and TP2 (**Figures 6B, C**).

### Functional analysis of degranulation and activation markers in neutrophil subpopulations

**Figure 7:**
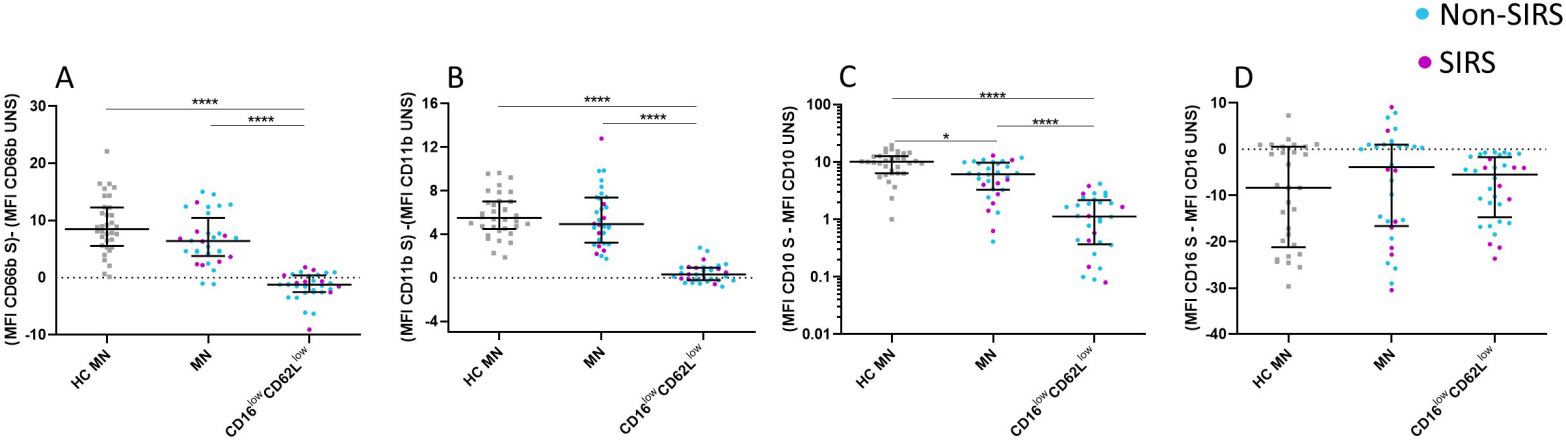
Alterations in CD marker expression among specific neutrophil subpopulations following *Staphylococcus aureus* stimulation. CD16^low^CD62L^low^ neutrophils demonstrate significant functional impairments, as evidenced by their reduced degranulation and activation capacity. These neutrophils exhibit a diminished change in CD66b **(A)**, CD11b **(B)**, and CD10 **(C)** expression compared to mature neutrophils (MN) following *Staphylococcus aureus* (SA) stimulation, as observed in both patients and healthy controls. In contrast, CD16 expression is comparable between CD16^low^CD62L^low^ neutrophils and mature neutrophils after SA stimulation **(D)**. Data were non-normally distributed (Shapiro-Wilk test), and statistical analysis was conducted using the Kruskal-Wallis test with Dunn’s correction for multiple comparisons (all vs. all); *p ≤ 0.05, ****p ≤ 0.0001. Sample sizes: TP1: N = 29 healthy controls, 24 Non-SIRS patients, and 8 SIRS patients. S = stimulated; UNS = unstimulated.

Given that CD16^low^CD62L^low^ neutrophils demonstrated a significantly reduced capacity for both oxidative burst and phagocytosis, we subsequently evaluated whether this subpopulation also exhibits alterations in activation and degranulation. Functional assays were performed to evaluate the degranulation capacity of mature neutrophils and CD16^low^CD62L^low^ neutrophils by measuring changes in CD66b expression following stimulation with SA. Upon stimulation, MN exhibited a significant increase in CD66b expression, while CD16^low^CD62L^low^ neutrophils did not exhibit an increase in CD66b expression (**Figure 7A**). Activation levels were further assessed by analyzing CD11b expression, which typically upregulates on the neutrophil surface following stimulation. MN from trauma patients displayed a CD11b expression increase comparable to that observed in healthy controls, whereas CD16^low^CD62L^low^ neutrophils failed to show any significant upregulation (**Figure 7B**).

The expression of CD10, another activation marker, was also examined. Similar to the trend observed with CD66b, MN exhibited an increase in CD10 expression upon stimulation with SA, whereas CD16^low^CD62L^low^ neutrophils remained unresponsive. Notably, MN from trauma patients demonstrated a lower upregulation of CD10 compared to MN from healthy controls (**Figure 7C**). Lastly, CD16 expression was analyzed, revealing consistent levels across both neutrophil populations, with no significant differences compared to MN from healthy controls, following stimulation with SA. (**Figure 7D**).

### Mitochondrial functionality in neutrophil subpopulations

Given the low CD16 expression and the reduced functional capacity for phagocytosis and ROS production observed in CD16^low^CD62L^low^ neutrophils, we investigated whether this population retained mitochondrial functionality using mitochondrial functional assays. Mitochondrial content was assessed using the Mitotracker Green probe, mitochondrial membrane potential with the Mitotracker Red probe, and mitochondrial superoxide production with the MitoSOX probe (14).

**Figure 8:**
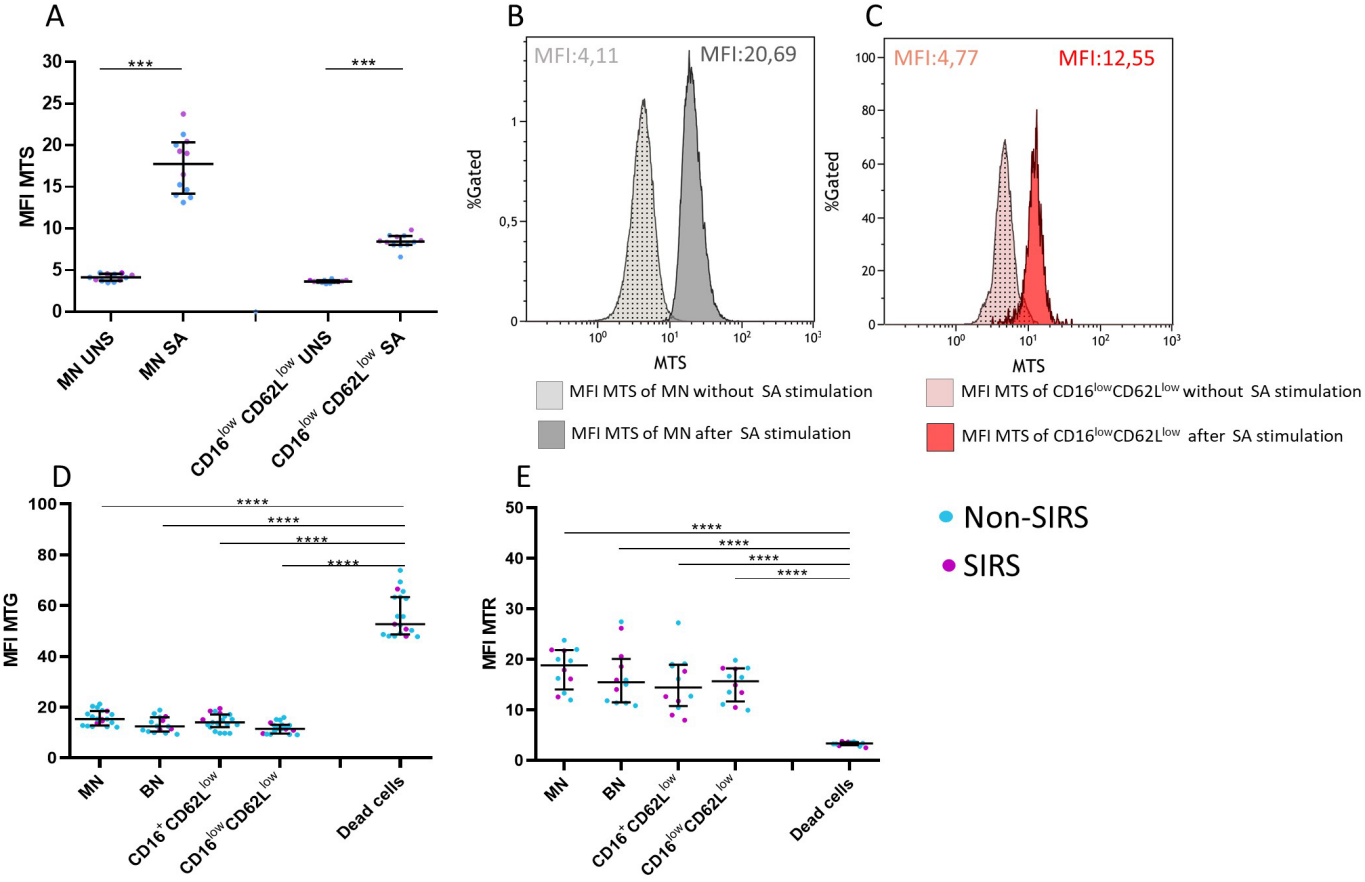
Mitochondrial characteristics of neutrophil subpopulations. CD16^low^CD62L^low^ neutrophils exhibit reduced production of mitochondrial superoxide (MTS) compared to mature neutrophils (MN) **(A)**. **(B)** Histogram showing mitochondrial superoxide production after *Staphylococcus aureus* (SA) stimulation in MN neutrophils. **(C)** Histogram showing mitochondrial superoxide production after SA stimulation in CD16^low^CD62L^low^ neutrophils. **(D)** All examined neutrophil subpopulations in Non-SIRS and SIRS patients exhibit comparable mitochondrial content (MFI MTG) and mitochondrial membrane potential (MFI MTP) **(E)**, which differ from those of dead cells. Data were non-normally distributed (Shapiro-Wilk test), and statistical analysis was conducted using the Kruskal-Wallis test with Dunn’s correction for multiple comparisons (all vs. all); *p ≤ 0.05, **p ≤ 0.01, ***p ≤ 0.001, ****p ≤ 0.0001. Sample sizes: N = 7 Non-SIRS patients, 5 SIRS patients. S = stimulated; UNS = unstimulated.

Compared to MN, CD16^low^CD62L^low^ neutrophils exhibited a comparable mitochondrial content and preserved mitochondrial membrane potential, both clearly distinct from dead cells (**Figures 8D, E**). However, upon stimulation with SA, CD16^low^CD62L^low^ neutrophils demonstrated a significantly reduced capacity for mitochondrial superoxide production (**Figures 8A–C**).

## Discussion

In this study, we demonstrate that neutrophils from trauma patients exhibit significantly altered counts and proportions of neutrophil subpopulations, with an increased representation of banded neutrophils, a trend that is further accentuated in patients who developed SIRS. The mechanisms driving this shift in neutrophil subsets remain unclear, but it is plausible that severe tissue injury and systemic inflammation contribute to the observed changes.

Trauma triggers the activation and recruitment of neutrophils from the bone marrow, leading to their accumulation both at the site of inflammation and in peripheral blood (6). This phenomenon is reflected in elevated neutrophil counts observed in trauma patients, which align with our clinical observations (15). Notably, the increase involves not only mature neutrophils but predominantly banded neutrophils (**Figure 2**). The early presence of banded, segmented, and hypersegmented neutrophils—characterized by CD16/CD62L expression and associated with nuclear segmentation—has been linked to late-onset infectious complications and progression to multi-organ failure during hospitalization in major trauma patients (8, 9). Our study confirmed a significant increase in the percentage and absolute counts of BN in trauma patients, with the increase being more pronounced in those with non-infectious SIRS (**Figure 2**, **Supplementary Figure 1**). While standard hematology analyzers often fail to detect these immature neutrophils, they can be identified using microscopy or flow cytometry, where they exhibit a CD16^low^CD62L^high^ phenotype as described by Hellebrekers et al. (9). Nevertheless, consistent with previous studies, our findings using hematology analyzers did not show a significant rise in immature neutrophils in SIRS patients compared to those without SIRS. The immaturity of CD16^low^CD62L^high^ neutrophils we confirmed not only by their reduced expression of CD16 but also by lower expression of CD10 and CD11b (**Figure 4**), as is typically described for immature neutrophils (16).

We show that patients with non-infectious SIRS exhibit increased serum lactate levels compared to Non-SIRS patients (**Table 1**). Furthermore, our data highlight that serum lactate levels at admission have the strong association with the proportion of banded neutrophils (**Table 2**). Elevated lactate, a marker of metabolic stress and tissue hypoperfusion, reflects the severity of cellular injury and hypoxia and has been widely recognized as a predictor of trauma severity and early clinical deterioration (17, 18). Similarly, elevated CK levels, an indicator of muscle damage, correlate with the percentage of banded neutrophils, particularly in patients with traumatic SIRS (**Table 2**). CK levels are also associated with complications such as acute kidney injury, particularly in cases of rhabdomyolysis, a condition linked to increased mortality (19). The observed correlations between banded neutrophils and lactate or CK levels suggest that metabolic stress and muscle injury drive an increased demand for immature neutrophils, accompanied by a corresponding reduction in mature neutrophils. This phenomenon reflects the emergency granulopoiesis that occurs in response to trauma, as previously described (6). Interestingly, our findings support the hypothesis that lactate levels play a pivotal role in driving neutrophil mobilization from the bone marrow. This mechanism has been previously described, with lactate shown to influence emergency granulopoiesis and neutrophil release (20). These results point to the role of metabolic and tissue stress in shaping the neutrophil response to trauma.

Importantly, our data further reveal significant correlations between banded neutrophil levels, Injury Severity Scores, and Trauma and Injury Severity Scores (**Table 3**). While ISS reflects the anatomical severity of injuries, TRISS combines injury severity with physiological parameters and demographic factors to predict survival outcomes. Notably, TRISS scores also correlated with banded neutrophil proportions at TP2, approximately four days after admission, indicating a sustained association between neutrophil dynamics and trauma severity.

As inflammation progresses, regulatory mechanisms are crucial to restore homeostasis after the clearance of damaged tissues. Hypersegmented CD16^high^CD62L^low^ neutrophils may contribute to this process by regulating pro-inflammatory T-lymphocyte activity through Mac-1 receptors, as described by Pillay et al. (8). In our study, we observed an increase in hypersegmented neutrophils in Non-SIRS patients at TP1 and in SIRS patients at TP2 (**Figure 2**; **Supplementary Figure 1**). The negative correlation between hypersegmented neutrophils and lactate levels, (**Table 2**), along with their lower abundance in SIRS patients at TP1, suggests an impaired immune response regulation in these individuals. By TP2, particularly in SIRS patients, we noted a rise in hypersegmented neutrophils accompanied by a decline in banded neutrophils (BN). This shift may indicate the onset of a successful return to homeostasis. However, further studies are needed to confirm this hypothesis and fully elucidate the role of hypersegmented neutrophils in immune regulation following trauma and non-infection SIRS.

The role of CD16^low^CD62L^low^ neutrophils remains unclear. These cells display impaired functional properties, including reduced phagocytosis and oxidative burst (**Figures 5**, **6**), likely reflecting reduced expression of the markers CD10, CD11b, CD181, and CD182 (**Figure 4**). Reduced expression of CD181 and CD182, key IL-8 receptors, indicates reduced chemotactic capacity (21). In line with this, Cortjens et al. (10) reported that CD16^low^CD62L^low^ neutrophils were restricted to peripheral blood but not found in bronchoalveolar lavage fluid, suggesting impaired migration. This population was further characterized by low expression of CD11b, which plays a crucial role in neutrophil migration and phagocytosis and T-cell regulation (8). Importantly, despite the loss of CD16, a characteristic commonly associated with apoptotic neutrophils (22), our mitochondrial assays confirmed that these cells were viable and non-apoptotic. Apoptosis is typically initiated by outer mitochondrial membrane permeabilization, which is closely linked to a loss of mitochondrial membrane potential (23). CD16^low^CD62L^low^ population demonstrated a mitochondrial membrane potential comparable to that of mature neutrophils (**Figure 8**).

CD16^low^CD62L^low^ neutrophils have previously been identified in infants with severe viral or bacterial infections, where they have been shown to include myelocytes and metamyelocytes (10). This population was characterized by decreased expression of CD11b and CD54 and increased expression of CD63 and CD66b. In infected infants, the number of neutrophil progenitors gradually increased between the third and sixth day of infection, a trend similar to what we observed in our cohort of trauma patients. However, unlike the pediatric group, we did not observe elevated CD66b expression in trauma patients, which may be attributed to differences in age and the nature of the underlying conditions in the two studied populations.

In addition to their surface expression patterns, the functional roles of CD16 and CD62L provide further insight into the significance of the CD16^low^CD62L^low^ neutrophil subset in trauma. CD62L (L-selectin) is a key adhesion molecule that facilitates neutrophil attachment and rolling along the endothelium during the early stages of migration into inflamed tissues. Beyond adhesion, CD62L also participates in outside-in and inside-out signaling pathways that regulate neutrophil activation, arrest, and transmigration (24, 25). Upon ligation, it triggers intracellular signaling cascades essential for neutrophil function, including calcium influx, tyrosine phosphorylation, MAPK (ERK, JNK) activation, and production of reactive oxygen species that collectively promote gene expression, cytoskeletal reorganization, and microbial killing (26). These responses also enhance the activity of integrins, particularly β1, and support Rac-mediated actin rearrangements that are critical for firm adhesion and transmigration (26). The downregulation of CD62L in the CD16^low^CD62L^low^ subset suggests disruption of the aforementioned key signaling and adhesion processes, implying a reduced capacity for endothelial interaction and tissue infiltration. Similarly, decreased CD16 expression may contribute to impaired phagocytosis and oxidative burst (27), consistent with the functional deficits observed in this population.

In our cohort, a significant increase in the percentage of CD16^low^CD62L^low^ neutrophils was observed around four days post-trauma, possibly indicating a subsequent phase of the inflammatory response in trauma patients. Despite their impaired effector functions, the expansion of this subset may reflect a regulatory mechanism within trauma-induced systemic inflammatory response syndrome (SIRS). Their accumulation in the circulation, combined with limited oxidative activity, reduced phagocytosis, and poor tissue migratory capacity, may serve to dilute the pool of highly reactive neutrophils, thereby moderating the overall oxidative and proteolytic burden characteristic of SIRS (28).

Our study demonstrates that all four neutrophil populations, defined by the expression of CD16 and CD62L, exhibit stable and characteristic surface marker expression of CD10, CD11b, and CD66b (**Supplementary Figure 2**). This expression remains unchanged over time and is comparable between patients with SIRS and those without SIRS. The stability of these surface markers suggests that the fundamental biological activity of these neutrophil populations is not influenced by the presence or absence of an acute inflammatory condition such as SIRS. Furthermore, the relative proportions of banded neutrophils, mature neutrophils, hypersegmented neutrophils and CD16^low^CD62L^low^ neutrophils in trauma and SIRS may indicate their role as consistent indicators of a patient’s current pro-inflammatory state.

However, this study has certain limitations. Due to the loss of CD62L expression upon neutrophil activation, we were unable to assess the functional capacity of banded and hypersegmented neutrophils following stimulation with *Staphylococcus aureus* or *E. coli* (**Figures 5**, **6**). Future studies should focus on identifying alternative activation markers that remain stable under short-term stimulation conditions, thereby facilitating functional analysis of neutrophil subpopulations. Additionally, we lack morphological confirmation of the CD16^low^CD62L^low^ population; however, its phenotypic similarity and temporal occurrence align with previous descriptions of progenitor neutrophils.

## Conclusion

In conclusion, analysis of neutrophil subpopulations defined by CD16 and CD62L expression provides a comprehensive understanding of neutrophil activation and function in trauma patients. Increased proportion of banded neutrophils correlates with systemic inflammation and SIRS, whereas hypersegmented neutrophils may contribute to the resolution of inflammation. Monitoring of these subpopulations could help identify trauma patients at risk for developing non-infectious SIRS. Furthermore, we have shown that CD16^low^CD62L^low^ neutrophils exhibit reduced functional properties, including impaired phagocytosis and oxidative burst. Neutrophil activation and recruitment during trauma can exacerbate tissue damage and prolong inflammation. However, the presence of CD16^low^CD62L^low^ neutrophils, characterized by their reduced capacity for ROS and associated NOS production, may serve as an anti-inflammatory factor. This hypothesis, however, requires further investigation in future studies.

## Data Availability

The raw data supporting the conclusions of this article will be made available by the authors, without undue reservation.

## Supplementary material

Supplementary Figure 1 Frequency of leukocyte and neutrophil subsets at TP1 and TP2. Frequency of leukocytes **(A)**, frequency of neutrophils **(B)**, and absolute neutrophil counts **(C)** in healthy controls, Non-SIRS patients, and SIRS patients at two time points: TP1 and TP2. Absolute counts of neutrophil subsets in healthy controls and both patient groups at TP1 and TP2: **(D)** mature neutrophils (MN), **(E)** banded neutrophils (BN), **(F)** CD16^high^CD62L^low^ neutrophils, and **(G)** CD16^low^CD62L^low^ neutrophils. Data were non-normally distributed (Shapiro-Wilk test), and statistical analysis was conducted using the Kruskal-Wallis test with Dunn’s correction for multiple comparisons (all vs. all); *p ≤ 0.05, **p ≤ 0.01, ***p ≤ 0.001, ****p ≤ 0.0001. Sample sizes: TP1: N = 39 healthy controls, 33 Non-SIRS patients, 16 SIRS patients; TP2: N = 31 healthy controls, 31 Non-SIRS patients, 13 SIRS patients.

Supplementary Figure 2 Phenotypic analysis of neutrophil subpopulations. Decreased expression of CD10 and CD11b on banded neutrophils (BN) at TP1. Cell surface expression of neutrophil markers in different neutrophil populations at TP1 and TP2, including **(A)** CD16, **(B)** CD62L, **(C)** CD66b, **(D)** CD11b, **(E)** CD10. CD16^low^CD62L^low^ neutrophils consistently exhibit low expression of all analyzed markers at both time points. Data were non-normally distributed (Shapiro-Wilk test), and statistical analysis was conducted using the Kruskal-Wallis test with Dunn’s correction for multiple comparisons (all vs. all); *p ≤ 0.05, ***p ≤ 0.001. Sample sizes: CD16, **(B)** CD62L, **(C)** CD66b, **(D)** CD11b, TP1: N = 39 healthy controls, 33 Non-SIRS patients, 16 SIRS patients; TP2: N = 31 healthy controls, 31 Non-SIRS patients, 13 SIRS patients; CD10, **(E)** TP1: N = 39 healthy controls, 23 Non-SIRS patients, 9 SIRS patients; TP2: N = 21 Non-SIRS patients, 7 SIRS patients.
