# Supplementary figures and images for: Pro-inflammatory role of neutrophils populations in trauma patients: monitoring neutrophil populations
Source: Front Immunol. 2025 Jul 8;16:1565606. doi: 10.3389/fimmu.2025.1565606 (PMC12280902; doi:10.3389/fimmu.2025.1565606)

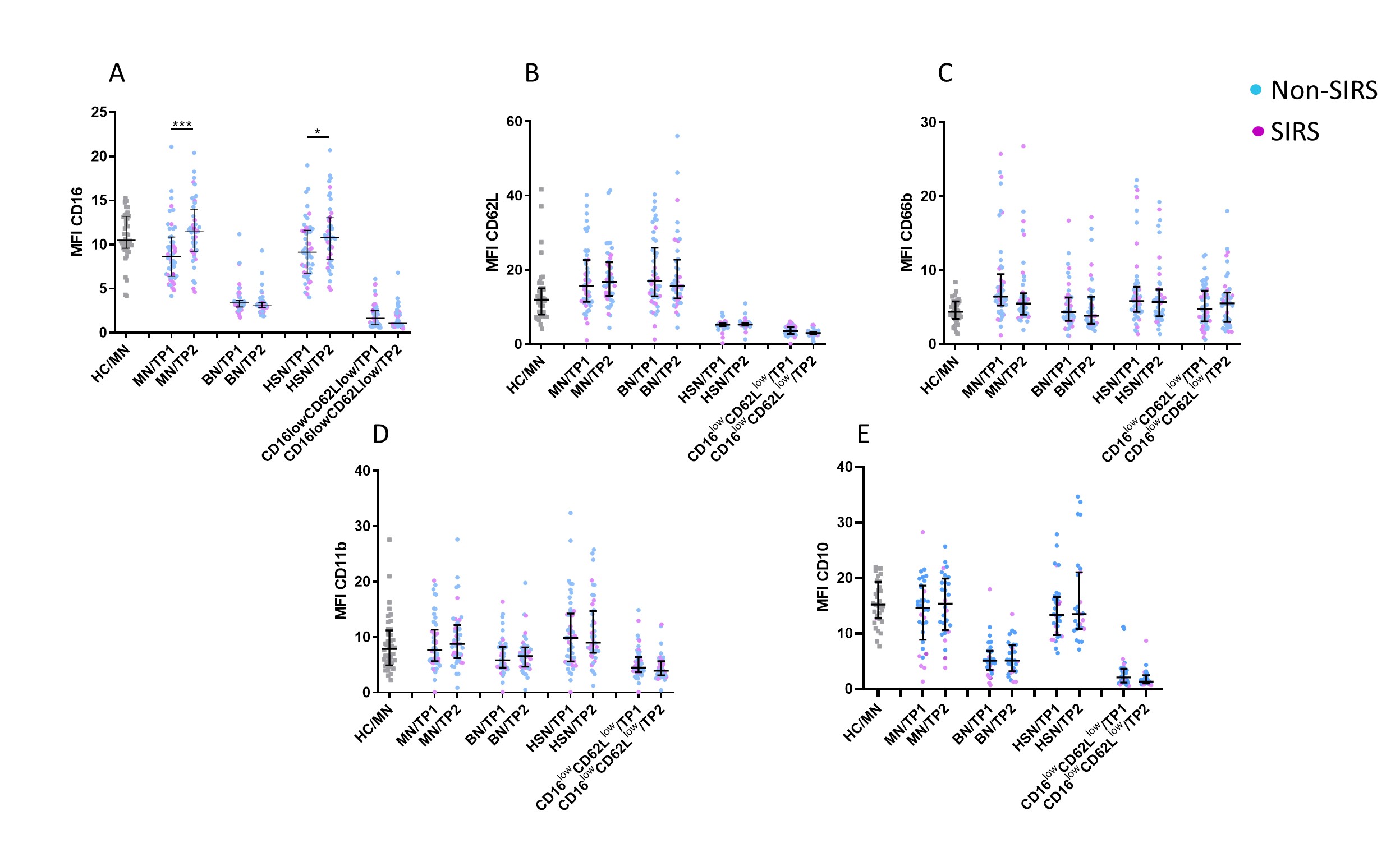

Supplement: Supplementary Figure 1 — Frequency of leukocyte and neutrophil subsets at TP1 and TP2. Frequency of leukocytes (A), frequency of neutrophils (B), and absolute neutrophil counts (C) in healthy controls, Non-SIRS patients, and SIRS patients at two time points: TP1 and TP2. Absolute counts of neutrophil subsets in healthy controls and both patient groups at TP1 and TP2: (D) mature neutrophils (MN), (E) banded neutrophils (BN), (F) CD16highCD62Llow neutrophils, and (G) CD16lowCD62Llow neutrophils. Data were non-normally distributed (Shapiro-Wilk test), and statistical analysis was conducted using the Kruskal-Wallis test with Dunn’s correction for multiple comparisons (all vs. all); *p ≤ 0.05, **p ≤ 0.01, ***p ≤ 0.001, ****p ≤ 0.0001. Sample sizes: TP1: N = 39 healthy controls, 33 Non-SIRS patients, 16 SIRS patients; TP2: N = 31 healthy controls, 31 Non-SIRS patients, 13 SIRS patients. [file Image1.jpeg]

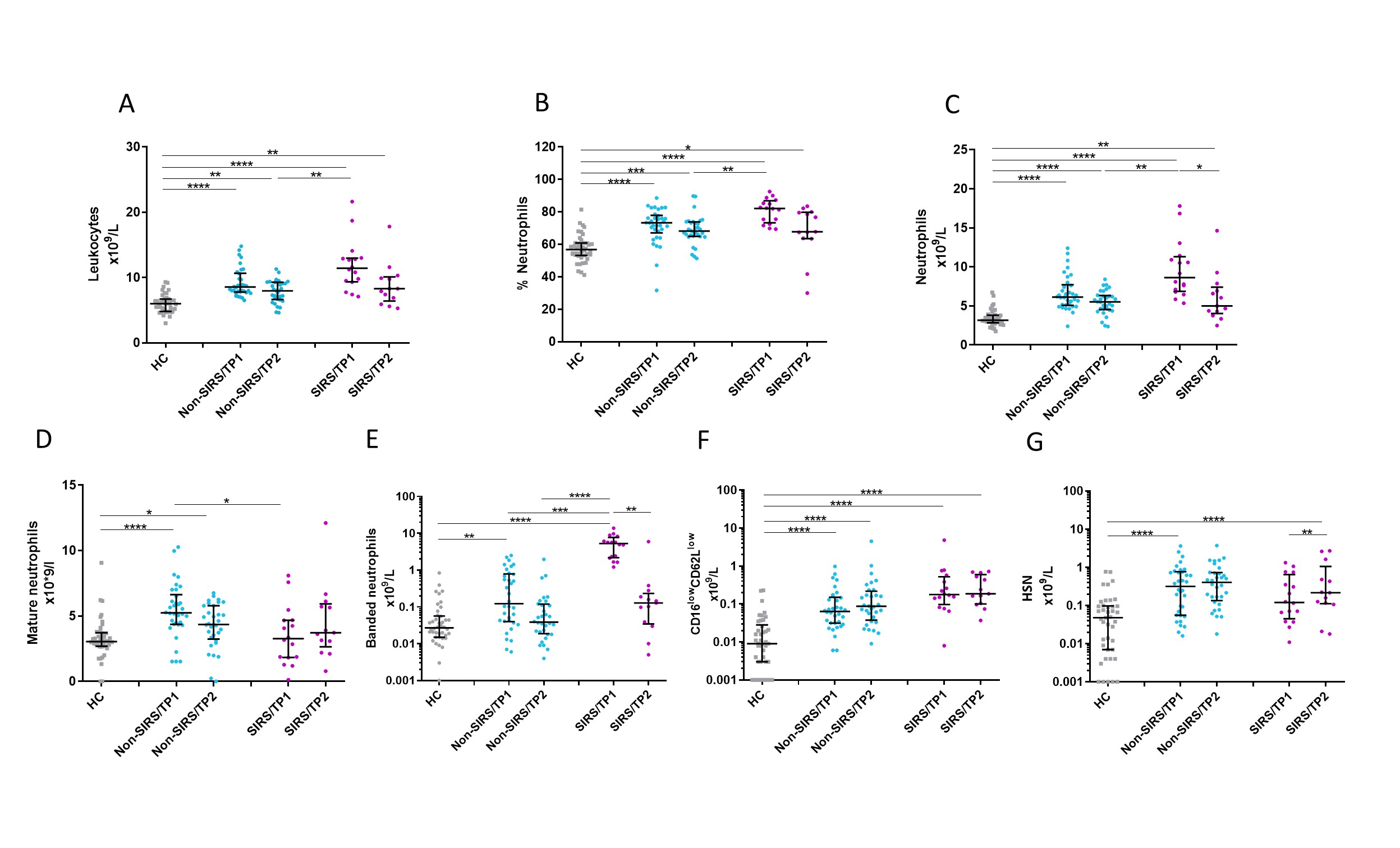

Supplement: Supplementary Figure 2 — Phenotypic analysis of neutrophil subpopulations. Decreased expression of CD10 and CD11b on banded neutrophils (BN) at TP1. Cell surface expression of neutrophil markers in different neutrophil populations at TP1 and TP2, including (A) CD16, (B) CD62L, (C) CD66b, (D) CD11b, (E) CD10. CD16lowCD62Llow neutrophils consistently exhibit low expression of all analyzed markers at both time points. Data were non-normally distributed (Shapiro-Wilk test), and statistical analysis was conducted using the Kruskal-Wallis test with Dunn’s correction for multiple comparisons (all vs. all); *p ≤ 0.05, ***p ≤ 0.001. Sample sizes: CD16, (B) CD62L, (C) CD66b, (D) CD11b, TP1: N = 39 healthy controls, 33 Non-SIRS patients, 16 SIRS patients; TP2: N = 31 healthy controls, 31 Non-SIRS patients, 13 SIRS patients; CD10, (E) TP1: N = 39 healthy controls, 23 Non-SIRS patients, 9 SIRS patients; TP2: N = 21 Non-SIRS patients, 7 SIRS patients. [file Image2.jpeg]
